# Nutrition behaviour and compliance with the Mediterranean diet pyramid recommendations: an Italian survey-based study

**DOI:** 10.1007/s40519-019-00807-4

**Published:** 2019-11-08

**Authors:** Renata Bracale, Concetta M. Vaccaro, Vittoria Coletta, Claudio Cricelli, Francesco Carlo Gamaleri, Fabio Parazzini, Michele Carruba

**Affiliations:** 1 Department of Medicine and Sciences for Health, Molise University, Campobasso, Italy; 2 Fondazione Censis, Rome, Italy; 3 Società Italiana di Medicina Generale e delle Cure Primarie Firenze, Florence, Italy; 4 Ordine dei Farmacisti delle Province di Milano, Lodi e Monza Brianza, Milan, Italy; 5 Dipartimento di scienze Cliniche e di Comunità, Università di Milano, Milan, Italy; 6 Department of Medical Biotechnology and Translational Medicine, Center for the Study and Research on Obesity, University of Milan, Milan, Italy

**Keywords:** Compliance, Food pyramid, Italy, Lifestyle, Mediterranean diet, Nutrition

## Abstract

**Purpose:**

Adopting a Mediterranean-like dietary pattern may help in preventing several chronic diseases. We assessed the eating behaviour and compliance with the Mediterranean diet pyramid recommendations in Italy.

**Methods:**

This is a cross-sectional study conducted in subjects aged ≥ 20 years. A 14-question survey based on the updated Mediterranean diet pyramid was launched online from April 2015 to November 2016. At test completion, a personalized pyramid displaying the possible deficiencies and/or excesses was generated, that could be the basis to plan diet and lifestyle modifications.

**Results:**

Overall, 27,540 subjects completed the survey: the proportion of females (75.6%), younger subjects (20.7%) and people with a University degree (33.1%) resembled those of the Italian population of Internet users rather than of the general population. 37.8% of participants declared a sedentary lifestyle, including 29.6% of those aged 20–29 years. A lower-than-recommended intake of all food categories included in the Mediterranean diet pyramid, along with excess of sweets, red and processed meats, emerged, that may affect health in the long term. Low adherence to recommendations was observed especially among females and older people. Notably, a discrepancy surfaced between the responders’ perceived and actual behaviour toward the regular consumption of fruits and vegetables (81.8% vs 22.7–32.8%, respectively).

**Conclusions:**

The nutritional habits and lifestyle of Italian participants are poorly adherent to the Mediterranean diet recommendations. The personalized pyramid tool may help in raising the awareness of individuals and their families on where to intervene, possibly with the support of healthcare professionals, to improve their behaviour.

**Level of evidence:**

Level V, cross-sectional descriptive study.

**Electronic supplementary material:**

The online version of this article (10.1007/s40519-019-00807-4) contains supplementary material, which is available to authorized users.

## Introduction

Exhaustive evidence has established the central role of a healthy lifestyle, based on a balanced and diverse diet and on adequate physical activity, in preventing several chronic diseases, known to impose a substantial burden on healthcare systems and communities.

In particular, a number of studies have shown that adoption of a Mediterranean-like dietary pattern [1] improves the health status [2], decreases morbidity and mortality [3] and reduces the total lifetime costs [2]. Benefits have been demonstrated in terms of decreased risk of cardiovascular diseases (CVD) [4–6], obesity [7], metabolic syndrome [8, 9] and type-2 diabetes [10–13], as well as of certain types of neurodegenerative diseases and late-life cognitive disorders [14–17] and cancers [18–22]. Moreover, in the elderly, such dietary pattern has been reported to prolong survival [23] and reduce bone loss in subjects with osteoporosis supplemented with vitamin D [24, 25].

In the last decades, however, we have witnessed a change in dietary and lifestyle habits, that has led to the loss of the traditional Mediterranean diet pattern. Factors accounting for this shift include globalization, more poverty and sedentariness and increased intake of sugars and of energy-dense and processed foods. This is particularly relevant in Countries like Italy, where diabetes is rising (due to population aging, early diagnosis and prolongation of patient survival), and the rate of childhood obesity, although decreasing [26, 27], is one of the highest in Europe, with one child in five being obese [28]. As a consequence, the risk of adult obesity, diabetes and CVD later in life is expected to increase, rising a serious public health concern.

In this context, gaining insights into the actual nutrition habits of the Italians is the first step to plan simple and effective strategies aimed at improving diet and lifestyle or, where requested, to provide nutrient supplementation.

Here, we describe the nutrition behaviour of a large Italian population and their compliance with the Mediterranean diet pyramid recommendations [1].

## Methods

### Study design and participants

This population-based, cross-sectional study was undertaken to collect information about the nutrition behaviour of Italian subjects aged ≥ 20 years from across the Country. A 14-question survey was designed on the basis of the updated food pyramid of the Mediterranean diet, which establishes daily, weekly and occasional guidelines to follow a healthy and balanced diet [1]. Although not validated, the questionnaire employed was developed on the food frequency questionnaires used in published studies [1].

The survey was launched online on the Italian website www.curarelasalute.com, from April 2015 to November 2016. The venture was publicized mainly by pharmacists, informed through newsletters, activities of media relations on trade magazines and during events dedicated to pharmacists and nutritionists (e.g. Cosmofarma, Farmacista Più and Pianeta Nutrizione). Moreover, pharmacies employed leaflets and informative materials distributed onsite, post on Facebook and activities of media relations on consumer-targeted magazines to publicize the website.

The survey collected demographics, general information on the lifestyle and nutrition habits, and information on the number of servings of each food category included in the Mediterranean pyramid (for the corresponding quantities in g/day refer to [29] and [30]. The complete questionnaire is provided as Supplementary Table 1. At test completion, a personalized pyramid was generated, displaying the possible food excesses and deficiencies. Moreover, a suggestion was made to discuss the resulting pyramid with the general practitioner (GP) or pharmacist to adopt measures to improve the individual’s nutrition.

Participation was voluntary and anonymous and completing the survey was accepted as consent by the participants.

### Statistical analysis

Qualitative variables were expressed as absolute and relative frequencies; comparisons between groups were made by the Chi-square test. A *p* value of < 0.05 was considered as statistically significant. All analyses were conducted using IBM SPSS Statistics v25.

## Results

### Participant characteristics

Overall, 27,540 subjects (females: 75.6%) completed the survey. The main demographic characteristics of the overall participant population are summarized in Table 1. Based on gender and age distribution, the sample population reflected the population of the Italian Internet users (i.e., 61.4% of people older than 20 years [31]) rather than fully represent the general Italian population, particularly with regard to the proportion of females, younger subjects and higher level of education (Table 1 and data not shown).

**Table 1:** Demographics of the responders and comparison with the population of Internet users in Italy

Characteristic	Responders *N* = 27,540	Internet users^a^
Gender		
Females	75.6	47.6
Males	24.4	52.4
Age (years)		
20–29	20.7	18.6
30–39	19.2	22.9
40–49	22.9	25.8
50–59	24.1	19.6
≥ 60	13.1	13.1
Education		
Low	16.2	24.7
High school—Professional	47.1	50.2
University	33.1	22.2
Other	3.5	3.0
Geographical area		
Northwest	35.6	26.7
Northeast	20.0	19.1
Center	17.0	20.0
South and the islands	27.5	34.2

Data are expressed as frequencies (*N*, %)

^a^Data from the Italian Institute of Statistics

### Lifestyle and nutrition habits

**Fig. 1:**
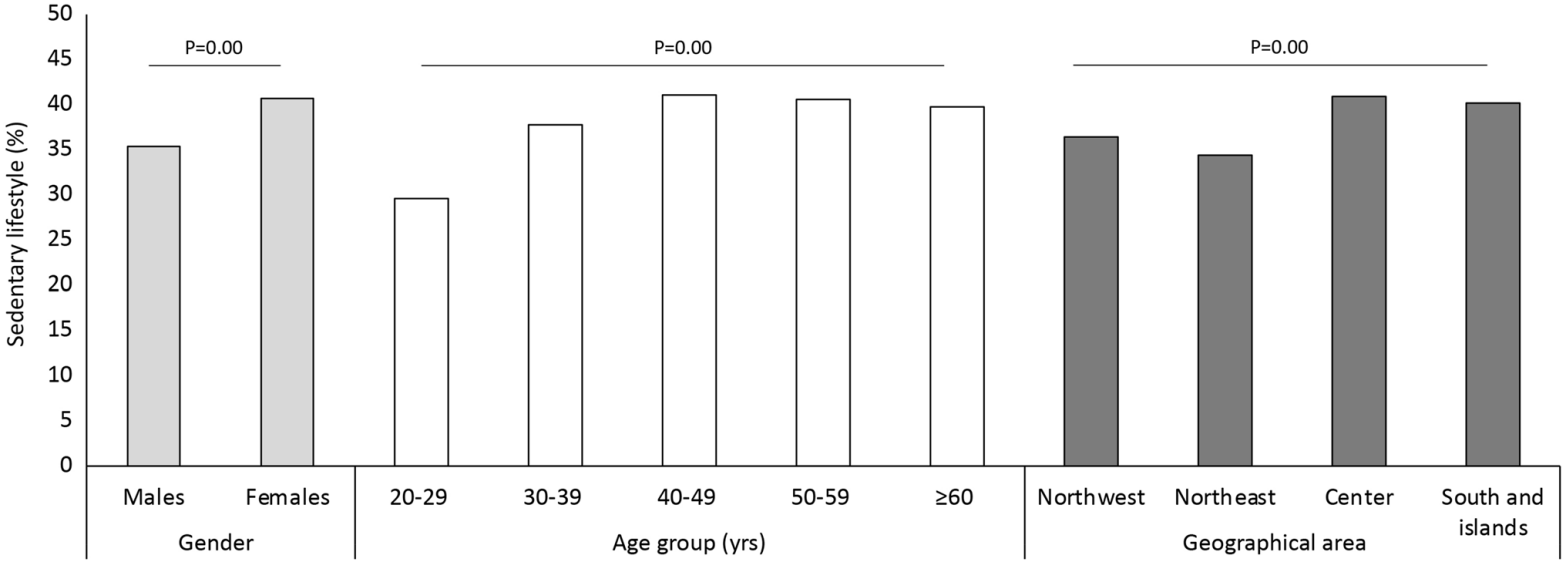
Sedentary lifestyle. Only the subgroups with statistically significant differences are displayed

Overall, 62.1% of responders declared to have an active lifestyle, doing physical activity (including walking, biking and taking the stairs rather than using the lift) at least twice a week. As shown in Fig. 1, sedentariness was significantly more common in older people (from 29.6% of those aged 20–29 years to 40% of those aged ≥ 50 year, *p* = 0.00), in females (40% vs 35.3% in males, *p* = 0.00) and in responders from Central and Southern Italy compared to those from the Northwest and the Northeast (40.9% and 40.2% vs 36.4% and 34.4%, respectively, *p* = 0.00).

**Fig. 2:**
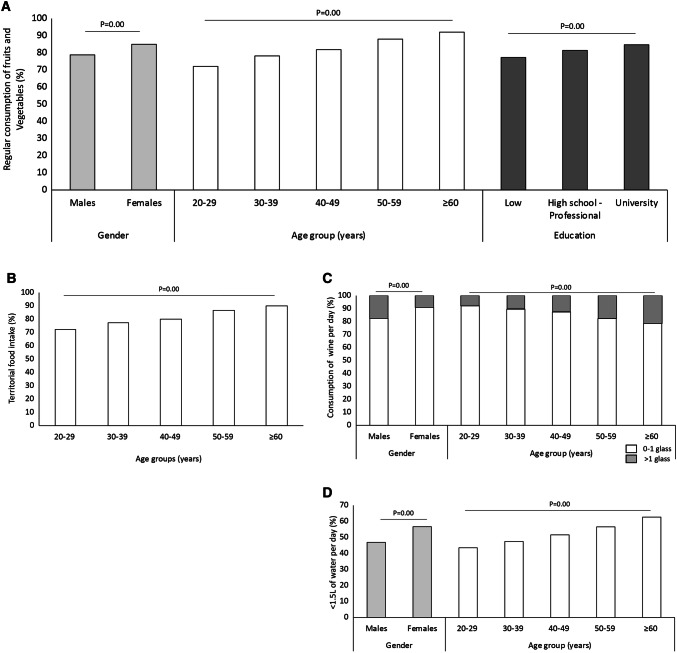
Nutrition habits in terms of regular intake of fruits and vegetables (**a**), territorial food (**b**), wine (**c**) and water (**d**). Only the subgroups with statistically significant differences are displayed

The regular consumption of fruits and vegetables was reported by 81.8% of participants and was significantly more common in females (85.0% vs 78.8% in males, *p* = 0.00) and increased with age (from 72.1% of those aged 20–29 years to 92.1% of those older than 60 years, *p* = 0.00) and education (from 77.3% of those with low education to 84.7% of those with a University degree, *p* = 0.00) (Fig. 2a). Moreover, 75.0% of the responders declared to consume fruits and vegetables of different colors (data not shown) and 80.7% that they had products from the territory, with local (i.e., typical of the tradition of the territory) food intake increasing with age (from 72.6% of those aged 20–29 years to 90.0% of those older than 60 years, *p* = 0,00) (Fig. 2b).

With regard to wine, 86.6% of the surveyed people declared to drink 0 or 1 glass of wine per day, while the remaining usually had more than one. The daily intake of wine was significantly lower among females (0–1 glass: 91.1% vs 82.6% of males; > 1 glass: 8.9% vs 17.4% in males *p* = 0.00) and in those aged ≥ 60 years (78.7%, vs 92.2% among those of 20–29 years, *p* = 0.00) (Fig. 2c).

Finally, 51.6% of the responders declared to drink at least 1.5 L of water daily; whereas, the remaining 48.4% had an insufficient intake. In particular, a level lower than recommended was reported significantly more frequently by females (56.7% vs 46.9% of males, *p* = 0.00) and with increasing age (from 43.5% of those aged 20–29 years to 62.7% of those older than 60 years, *p* = 0.00) (Fig. 2d).

### Compliance to the Mediterranean food pyramid recommendations

**Fig. 3:**
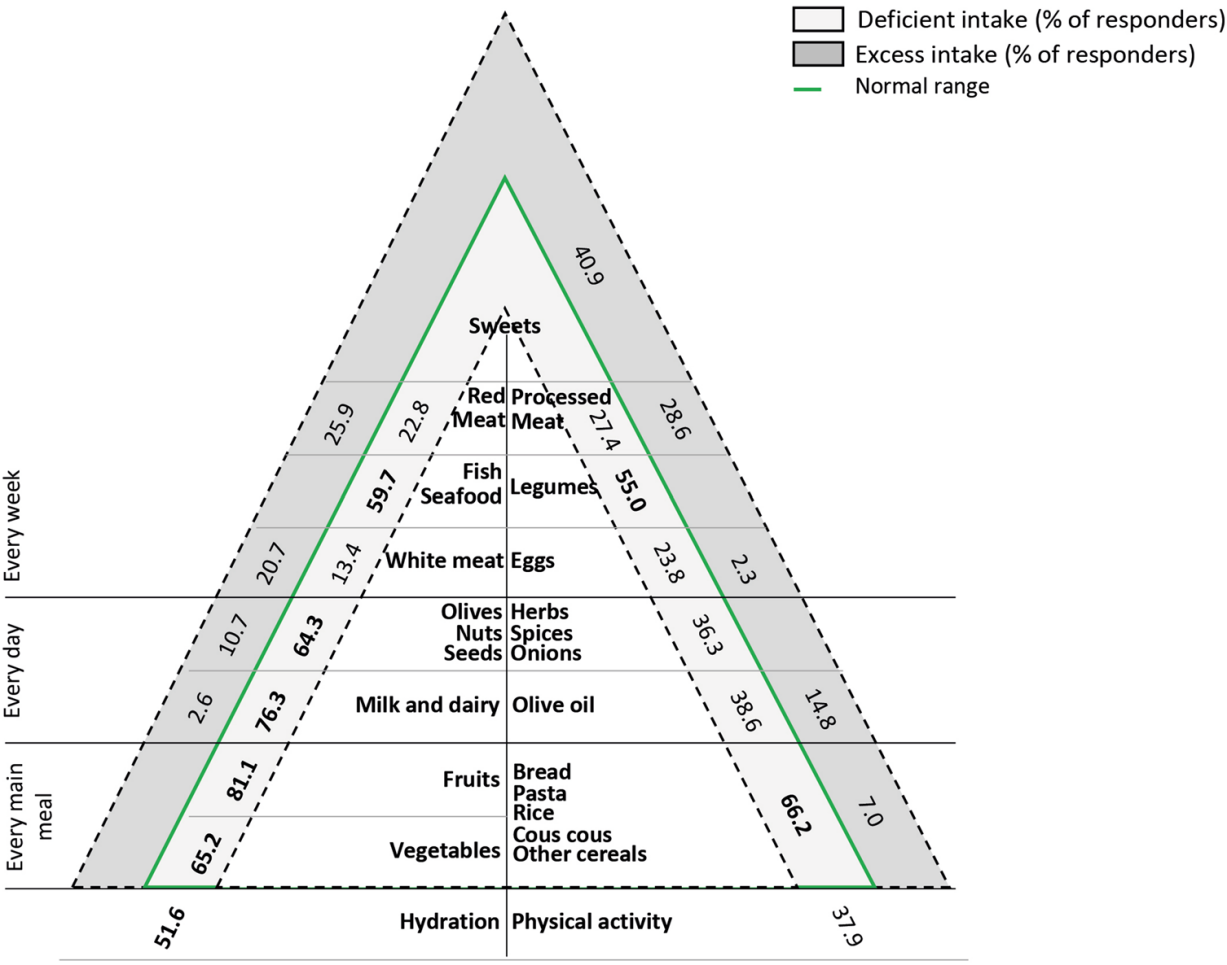
Food pyramid built on the participants’ responses to the questionnaire. The % of non-compliant responders in terms of excesses and deficiencies are displayed Modified from [1]

Next, to assess the compliance to the Mediterranean diet recommendations, responders were asked to indicate how many servings of each food group included in the pyramid they consumed at every main meal, daily, weekly or occasionally (Table 2). Deficiencies and excesses are detailed in the pyramid depicted in Fig. 3. Overall, deficiencies were observed for all food categories, while excesses were observed solely in some cases.

**Table 2:** Responder compliance to the Mediterranean food pyramid recommendations

	Compliant (%)	Non-compliant (%)
Every main meal		
Vegetables, ≥ 2 s	34.8	65.2
Fruits, ≥ 3 s	18.9	81.1
Bread, pasta etc., 4 s	26.8	73.2
Every day		
Milk and diary, 2–3 s	21.1	78.9
Nuts, 1 s	25.0	75.0
Herbs and spices	**63.7**	36.3
Olive oil, 3 s	46.6	53.4
Hydration	48.4	51.6
Every week		
White meat, 1–2 s	**65.9**	34.1
Fish and seafood, ≥ 2 s	40.3	59.7
Legumes, ≥ 2 s	45.0	55.0
Eggs, 1–4	**73.8**	26.2
Red meat, 1 s	**51.3**	48.7
Processed meat, 1 s	44.0	56.0
Sweets, ≤ 2 s	**59.1**	40.9
Physical activity	**62.1**	37.9

Compliance rates of at least 50% are indicated in bold

Data are expressed as %

*S* serving

**Fig. 4:**
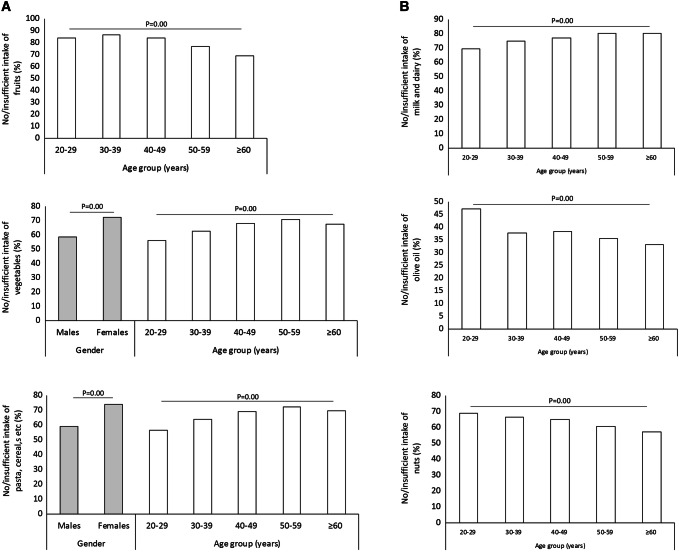

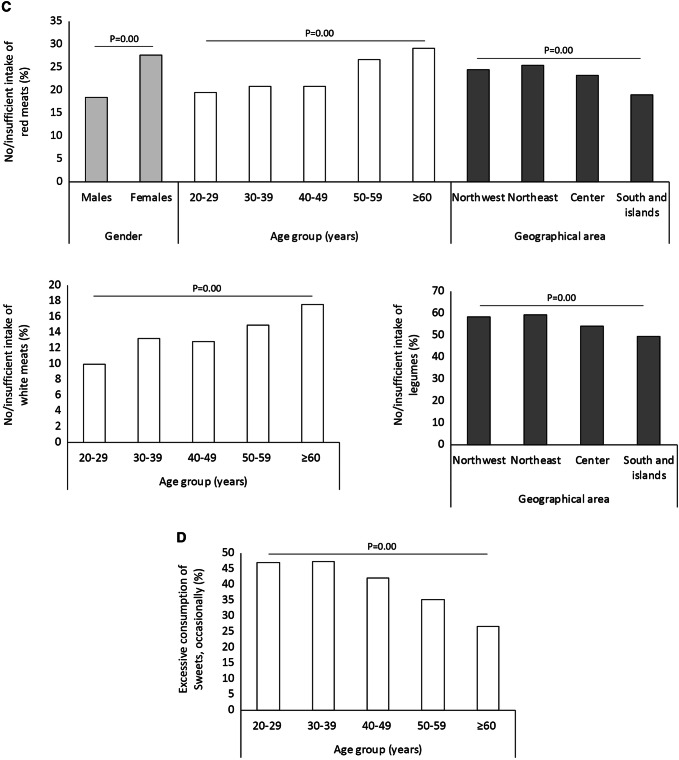
Intake of foods to be consumed at each main meal (**a**), daily (**b**) weekly (**c**) and occasionally (**d**). Only the subgroups with statistically significant differences are displayed

At each main meal, only 18.9%, 26.8% and 34.8% of the responders adhered to the recommendations on the servings of fruits, pasta and cereals and vegetables, respectively (which constitute the pyramid base) (Table 2). A lower-than-recommended intake of these foods was reported significantly more frequently by age and gender (Fig. 4a), except for fruits, consumed at a similar extent by males and females. Notably, a discrepancy was observed between the % of participants who had declared a regular intake of fruits and vegetables (i.e. 81.8), and those stating to actually consume a recommended amount of these foods (22.7% for fruits and 32.8% for vegetables; *p* = 0.00).

A low level of compliance was reported also for the foods to be consumed on a daily basis, except for herbs and spices (normal consumption in 63.7% of cases, vs 36.3% of no or infrequent intake) (Table 2). The lowest rate of compliance was observed for milk and dairy (21.1%) and nuts (25.0%) (Table 2). Consumption was mostly deficient and varied by age (Figs. 3, 4b).

Most of the responders declared a normal weekly consumption of animal protein-enriched foods: 51.3% for red meats, 65.9% for white meats and 73.8% for eggs (Table 2). A scarce intake of red meats was reported significantly more frequently among females (27.7% vs 18.4% of males), in Southern Italy (19.0% vs 25.4% in the Northeast) and with increasing age (from 19.6% in those aged 20–29 years to 29.1% in those older than 60 years), just like white meats (from 10.0% in those aged 20–29 years to 17.6% in those older than 60 years). In contrast, the weekly consumption of fish and seafood and of legumes was rather scarce (in 59.7% and 55.0% of responders, respectively; Table 2; Fig. 3), the latter being lower in Southern Italy (49.2% vs 59.2% in the Northeast) (Fig. 4c).

In the Mediterranean diet, sugar- and unhealthy fat-rich foods like sweets are allowed occasionally and in small amounts. In the present study, 59.1% of responders declared to have a normal consumption of sweets (52.9% of people between 20 and 29 years and 73.2% of those older than 60), whereas the remaining had excesses (Fig. 4d).

## Discussion

This population-based study provides a snapshot of the nutritional habits and lifestyle of the Italian Internet users participating in the survey between April 2015 and November 2016. The tool employed is the food pyramid based on the updated food pyramid of the Mediterranean diet [1].

Overall, the population participating in the study was more representative of the Italian Internet users rather than of the general population, as shown by the higher percentage of females, young subjects and those with a University degree. This result may be explained, at least partly, by the fact that the population of Internet users is younger than the general population, and, therefore, is more similar to the responders. Nevertheless, some interesting observations can be made. First, notwithstanding the clear interest of the responders in health, the younger age and the higher education level (adults with a high level of education are overrepresented in our sample population), nearly 40% of the participants declared to not conduct an active lifestyle. These data are in line with those collected by the Italian Institute of Statics, which, however, refer to a slightly different population (i.e., older than 6 years of age) [32]. It is not surprising that a significantly increased sedentariness was observed with increasing age, but the fact that 1 of 3 subjects between 20 and 29 years of age declared a sedentary behaviour is quite concerning. Indeed, sedentariness as well as suboptimal nutrition represents the modifiable risk factors for chronic diseases like obesity and type 2 diabetes [33], and adequate physical activity is recommended in the Mediterranean diet as a basic complement to nutrition for balancing the energy intake and maintaining a healthy body weight [1].

Although these data on the active lifestyle are in line with those observed in the general population (as reported by the Italian Institute of statistics [34]), the latter include also subjects between 3 and 20 years of age, who increase the rate of people with a non-sedentary behaviour and are instead excluded from the present study.

One of the main objectives of the study was to highlight possible food deficiencies and/or excesses. Accordingly, the overall picture emerged indicates a lower-than-recommended intake of all food categories included in the Mediterranean pyramid, together with some excesses that may impact on the subject’s health status later in life. First, with regard to wine, the overall consumption was low to moderate: it is worth noting that red wine contains flavonoids and resveratrol, known to play a role in the prevention of several diseases, including CDV and cancer [35–38]. Yet, it must be underlined that the International Agency for Research on Cancer has labeled alcohol as a Group-1 carcinogen, which indicates that there is sufficient evidence to suggest that exposure in humans is carcinogenic [39].

More than half of the responders (especially females and those aged > 50 years) reported an insufficient intake of water per day. The risk of this behaviour is dehydration, which has been associated with increased morbidity, mortality and also with a high economic burden, particularly in the elderly, in whom the frequent coexistence of comorbidities requiring polypharmacy may exacerbate this status [40–43].

In addition to these findings, more than half of the responders declared a scarce consumption of all the basic elements of the pyramid (i.e., fruits, vegetables and cereals). An inadequate intake of these foods, which are central elements of the Mediterranean diet, exposes the whole population to specific micronutrient deficiencies that may compromise health [44]. Indeed, these foods are an important source of vitamins, minerals and, above all, antioxidants, which protect the body from oxidative damage and, thus, from cellular aging [44, 45].

On the other hand, the excessive intake of some of the foods at the top of the pyramid, that indeed should be consumed only occasionally, surfaced. A higher-than-recommended intake of sweets, red meats and processed meats increases the risk of CVD, type 2 diabetes, obesity and other metabolic diseases, due to the high content of simple sugars and saturated fats [11, 46]. In this regard, the consumption of fish and olive oil should be increased, as they are rich in mono and polyunsaturated fats that prevent the development of CVD [47]. Moreover, the intake of milk and dairy should be optimized, because an adequate consumption of these products, enriched in calcium and vitamin D, may improve bone health [48].

When the compliance with the recommendations of the Mediterranean diet pyramid was evaluated by subgroups (i.e., gender, age, geographical area and education), a rather low adherence emerged mainly among females and the elderly, followed by people from different Italian regions. In all, the differences observed by geographical area are likely indicative of the different regional culinary traditions. An important health concern regards the insufficient consumption of milk and dairy products in females and older age groups, who are at high risk for osteoporosis. Indeed, a Mediterranean-like dietary pattern has been reported to reduce bone loss in subjects with osteoporosis supplemented with vitamin D [24, 25]. Moreover, vitamin D insufficiency has been implicated in a wide spectrum of conditions, ranging from musculoskeletal diseases to cancer and CVD [49–56], and supplementation of calcium and vitamin D has shown to be beneficial in reducing the risk of fractures [57, 58]. Nevertheless, the effects and appropriate regimen of calcium and vitamin D supplementation remain highly debated [50, 56, 59, 60].

Another result that deserves attention is the fact that females and older people consume insufficient amounts of several key foods included in the pyramid: in fact, this suggests a risk for suboptimal nutrition in children, because of the dependence from the parents’ choices [61]. This is especially worrying considering that the rate of childhood obesity in Italy, although declining, is still one of the highest in Europe [28].

Finally, the present study allowed to highlight possible discrepancies between the responders’ perceived and actual behaviour. Indeed, it is worth noting that 81.8% of participants had declared a regular consumption of fruits and vegetables, but only 22.7% and 32.8%, respectively, resulted compliant with the Mediterranean pyramid recommendations. This may be explained by the lack of information and awareness, by responders, on the correct intake of each food category.

The main limitation of the present study is the fact that responders do not fully represent the general Italian population. Moreover, the lack of data on BMI does not allow to correlate obesity with the compliance with the recommendations of the Mediterranean food pyramid. Finally, the food intake frequencies are self-reported, and this may lead to the actual misreporting of data. However, surveys similar to the one used here have been frequently employed.

On the other hand, the large sample size and the amount of information collected through the survey contribute to provide a reliable picture of the dietary pattern and lifestyle of participants.

From this population-based study, an overall low compliance of participants to the Mediterranean pyramid recommendations emerged, with several relevant food deficiencies and inconsistencies indicative of the low awareness of the subjects’ own nutrition habits and lifestyle. Importantly, the personalized pyramid generated following survey completion should serve to the responders for a self-evaluation of their dietary pattern, to possibly raise their family’s awareness on this matter, and to the healthcare professionals to plan simple intervention strategies to educate to a healthier behaviour and/or provide complete and balanced nutrient supplements. This is important in the long term, because of the positive impact that such measures may exert on public health in terms of chronic disease epidemiology and economic as well as social burden. In this context, GPs and pharmacists with their knowledge and expertise certainly play a key role and may support both health promotion and surveillance, which is required to monitor the situation and proactively evaluate the effectiveness of interventions.

## Electronic supplementary material

Below is the link to the electronic supplementary material.

Supplementary material 1 (DOCX 20 kb)
