# Supplementary material for: Nutrition behaviour and compliance with the Mediterranean diet pyramid recommendations: an Italian survey-based study
Source: Eat Weight Disord. 2019 Nov 8;25(6):1789–98. doi: 10.1007/s40519-019-00807-4 (PMC7581569; doi:10.1007/s40519-019-00807-4)
Supplement: Supplementary file 1 — Supplementary material 1 (DOCX 20 kb) [file 40519_2019_807_MOESM1_ESM.docx]

| **Questions** | **Answers** |
| --- | --- |
| *Demographics* |  |
| 1. Gender | a) F  b) M |
| 2. Age |  |
| 3. Education |  |
| 4. Geographical area |  |
| *Lifestyle and nutrition habits* |  |
| 5. Is your lifestyle active and do you practice sport at least twice a week? Do you often go for a walk/bike/ upstairs rather than using the elevator? |  |
| 6. Do you regularly consume seasonal fruits and vegetables? |  |
| 7. Do you prefer products from your territory? |  |
| 8. In your daily nutrition pattern, are fruits and vegetables of different colors included? |  |
| 9. How much wine do you drink daily? | a) 0-1 glass  b) >1 glass |
| 10. What size are your usual servings? | a) Scarce  b) Moderate  c) Abundant |
| 11. How much water do you drink daily? | a) <1.5 L  b) ≥1.5L |
| *Servings of each food group* |  |
| 12. How many servings of the following foods do you eat daily?  a) Fruits  b) Vegetables  c) Pasta, bread, rice, cereals, biscuits, potatoes and crackers  d) Milk and dairy  e) Olive oil  f) Nuts and seeds | a) 0; 1-2; ≥3  b) 0; 1; ≥2  c) <4; 4; >4  d) 0; 1; 2-3; >3  e) <2; 3; ≥4  f) <1; 1; ≥1 |
| 13. In your diet, do you use species, herbs, garlic or onionsas seasoning or to prepare food? | a) I do not use species, or I do very rarely, and I flavor foods with salt or salt-containing products  b) Yes, I use spices very often to reduce the salt intake in my diet |
| 14. How many servings of the following foods do you eat weekly? |  |
| a) Fish and seafoods | a) 0-1; ≥2 |
| b) White meat | b) 0; 1-2; >2 |
| c) Legumes | c) 0-1; ≥2 |
| d) Eggs | d) 0; 1-4; >4 |
| e) Processed meats | e) 0; 1; >1 |
| f) Red meat | f) 0; 1; ≥2 |
| g) Sweets | g) 0-2; >2 |

**Supplementary Table 1.** The 14-question survey launched online on the Italian website [www.curarelasalute.com](http://www.curarelasalute.com) from April to November 2015.
